# ALK-positive lung adenocarcinoma in a patient with rheumatoid arthritis with long-term treatment for organizing pneumonia: A case report

**DOI:** 10.1097/MD.0000000000032159

**Published:** 2022-12-09

**Authors:** Kazuhito Horie, Takanori Asakura, Keita Masuzawa, Hideki Terai, Sohei Nakayama, Yusuke Suzuki

**Affiliations:** a Department of Respiratory Medicine, Kitasato University Kitasato Institute Hospital, Tokyo, Japan; b Department of Clinical Medicine (Laboratory of Bioregulatory Medicine), Kitasato University School of Pharmacy, Tokyo, Japan; c Division of Pulmonary Medicine, Department of Medicine, Keio Cancer Center, School of Medicine, Keio University, Tokyo, Japan.

**Keywords:** anaplastic lymphoma kinase, lung cancer, organizing pneumonia, rheumatoid arthritis

## Abstract

Rheumatoid arthritis (RA) causes inflammation in various organs including the lungs. Pulmonary manifestations include inflammation of the pleura, vasculature, airway, and parenchyma, including interstitial lung disease (ILD). RA-organizing pneumonia (OP) is the third most common cause of RA-ILD. Cases of OP coexisting/complicated with lung cancer have been reported. Therefore, lung cancer can represent a diagnostic challenge, especially in patients with underlying pulmonary diseases including OP.

**Patient concerns:**

An 81-year-old woman with a 12-year history of RA-OP underwent multiple transbronchial lung biopsies (TBLBs), all of which resulted in no malignant findings. She was treated with prednisolone (PSL) depending on the deteriorated infiltrations. At admission, chest computed tomography (CT) images showed exacerbation of left S8 consolidation on chest CT. Additionally, her RA activity was exacerbated, and PSL dose was increased to 30 mg/day, which resulted in improved dyspnea and consolidation. Accordingly, PSL dose was gradually decreased. However, 6 months later, when PSL dose was 11 mg/d, due to a worsening of consolidation and the joint symptoms of RA, PSL dose was increased to 20 mg/d and tacrolimus 2 mg/d was administered. 3 months after the increase in PSL dose, dyspnea improved and PSL dose was reduced to 15 mg/d; however, she was admitted to our hospital because of low back pain.

**Diagnosis:**

Spinal magnetic resonance imaging showed bone metastases in the third and fifth lumbar vertebrae, and lung cancer was suspected as the primary tumor on CT.

**Interventions:**

TBLB was performed on the left B8 infiltrate, which showed no evidence of malignancy in the previous TBLB.

**Outcomes:**

Pathological examination of TBLB on the left B8 revealed an adenocarcinoma that was positive for anaplastic lymphoma kinase.

**Lessons:**

Physicians should be aware of the development of lung cancer in regions with OP, even after a partial response to corticosteroid therapy.

## 1. Introduction

Rheumatoid arthritis (RA) is the most prevalent type of collagen vascular disease, causing inflammation mainly in the synovial membranes of joints.^[1]^ It also causes inflammation in other organs, such as the skin, eyes, lungs, heart, and kidneys. Pulmonary lesions are common extra-articular lesions, which can affect up to 60% of patients with RA.^[2]^ Pulmonary manifestations include inflammation of the pleura, vasculature, airway, and parenchyma including interstitial lung disease (ILD). The incidence of lung cancer is higher in RA patients, likely owing to chronic lung inflammation.^[3]^ Additionally, RA patients with lung cancer, particularly those with ILD, showed lower survival rates than patients with lung cancer without RA.^[4]^

Organizing pneumonia (OP) is a disease response from the pulmonary reaction to harmful factors, including infections and exposure to toxic substances, drugs, and radiotherapy.^[5]^ Furthermore, OP can be observed in autoimmune disorders including RA.^[6]^ RA-OP is the third most common cause of RA-ILD, followed by usual and nonspecific interstitial pneumonia.^[2]^ OP can also be present in patients with lung cancer during their course (e.g., drug-induced ILD).^[7]^ Moreover, it has been reported that a single nodule or mass resected for patients with suspected lung cancer pathologically showed OP without malignancy.^[8]^ Some cases have been reported to be complicated/coexist with lung cancer.^[9]^ Therefore, lung cancer can represent a diagnostic challenge, especially in patients with underlying pulmonary diseases including OP.

Herein, we report the case of an elderly patient with anaplastic lymphoma kinase (ALK)-rearranged metastatic lung adenocarcinoma diagnosed during long-term treatment for recurrent RA-OP and successfully treated with alectinib.

## 2. Case presentation

**Figure 1.**
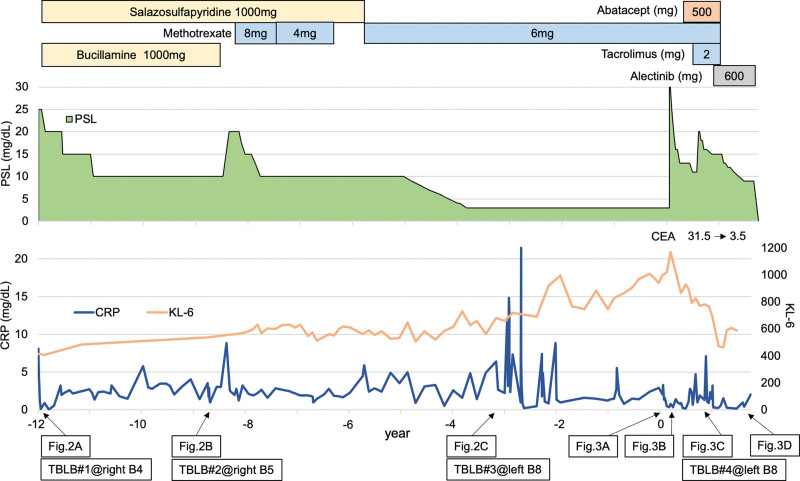
Clinical course of our patient. CEA = carcinoembryonic antigen, CRP = C-reactive protein, KL-6 = Krebs von den Lungen-6, PSL = prednisolone, TBLB = transbronchial lung biopsy.

**Figure 2.**
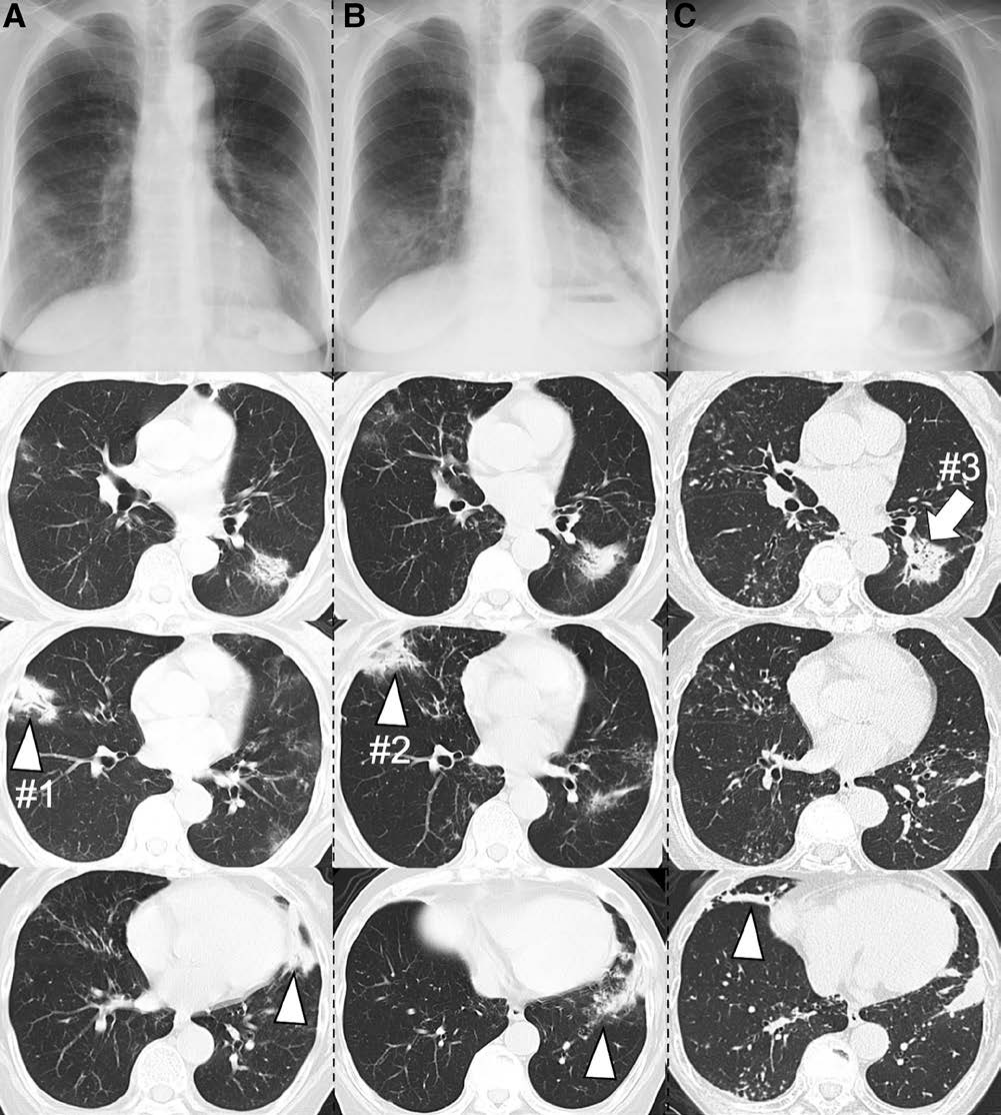
(A) Chest imaging 12 years before admission to our institution showed multifocal consolidations in the bilateral lungs. Transbronchial lung biopsy (TBLB) was performed targeting #1. (B) Chest imaging showed exacerbation of lung shadow after prednisolone (9 years before admission). TBLB was performed targeting #2. (C) 4 years before admission, the lung shadow had exacerbated again. TBLB from the left B8 (arrow, #3) showed no evidence of malignancy. Arrowheads indicate where infiltrates appeared and disappeared during the course.

Figure 1 shows the overall course summary. An 81-year-old woman was admitted to our hospital because of dyspnea. She had no history of smoking but had a 30-year history of RA and a 12-year history of OP. She had been treated with immunosuppressive drugs, including bucillamine, salazosulfapyridine, and methotrexate; however, upon admission, RA and OP were well-controlled with prednisolone (PSL) 3 mg/d. Twelve years prior to admission, a chest X-ray image showed bilateral lung shadows, and chest computed tomography (CT) images showed scattered consolidation in both lungs (Fig. 2A), which was refractory to antimicrobial therapy. Pathological examination of a transbronchial lung biopsy (TBLB) and brush through the right B4 bronchus (Fig. 2A#1) revealed infiltration of lymphocytes in the bronchial wall and granulomatous lesions in the alveoli, suggesting OP associated with autoimmune diseases. PSL 25 mg/d was administered, and its dose was gradually reduced; however, the patient experienced worsening dyspnea with exacerbation of the lung shadow (Fig. 2B). When the dose was reduced to 10 mg/d (9 years before admission), TBLB through right B5 (Fig. 2B#2) showed the same pathological results as those at the initial examination. Accordingly, PSL dose was increased to 20 mg/d, gradually reduced, and maintained at 3 mg/d. 7 months after the PSL dose was reduced to 3 mg/d (4 years before admission), the lung shadow exacerbated again (Fig. 2C), and pathological examinations of TBLB and brush through the left B8 bronchus (Fig. 2C#3) also revealed OP without evidence of malignancy. As there was no change in her symptoms, the same treatments were continued. RA was stable, except a post-cervical fusion for atlantoaxial subluxation.

**Figure 3.**
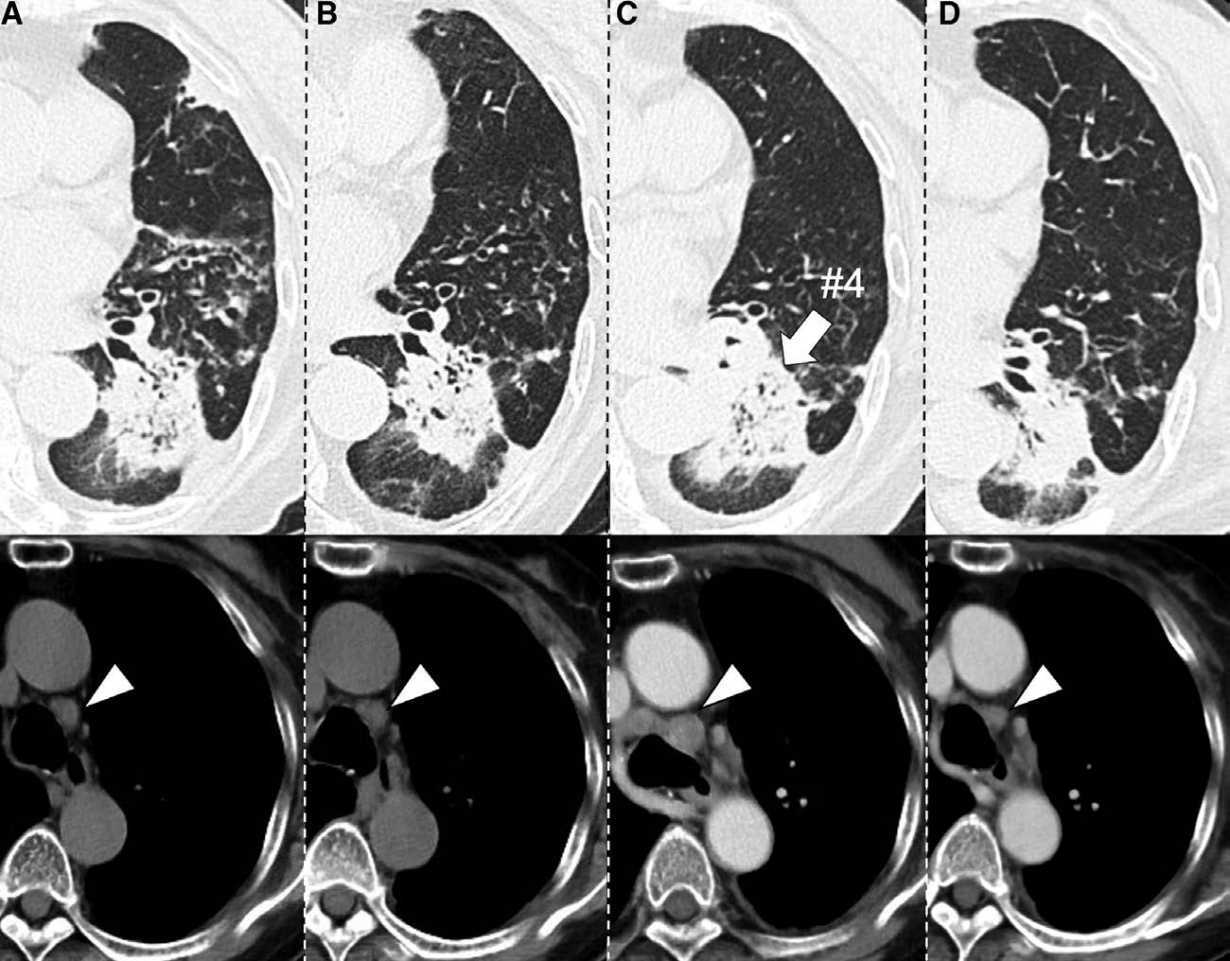
(A and B) At the current admission, chest computed tomography (CT) images showed an exacerbation of left S8 consolidation (A), and increasing PSL dose (30 mg/d) resulted in a partial improvement of consolidation (B). (C) CT images showed deterioration of consolidation at the time of adenocarcinoma diagnosis. TBLB performed targeting #4 (arrow). (D) 10 months of treatment with alectinib resulted in a partial response. Arrowheads indicate lymphadenopathy during the course. PSL = prednisolone, TBLB = transbronchial lung biopsy.

At the current admission, chest CT images showed exacerbation of the left S8 consolidation (Fig. 3A). Additionally, RA activity was exacerbated. The PSL dose was increased to 30 mg/d, which resulted in an improvement in dyspnea and consolidation (Fig. 3B). Consolidation persisted, but the symptoms of dyspnea improved, and PSL dose gradually reduced and abatacept 500 mg was administered for her RA. However, 6 months later, when PSL 11 mg/d was administered, due to worsening of consolidation and the joint symptoms of RA, PSL dose was increased to 20 mg/d and tacrolimus 2 mg was administered. 3 months after the increase in PSL dose, dyspnea improved and PSL dose was reduced to 15 mg; however, she was admitted to our hospital because of low back pain. Spinal magnetic resonance imaging revealed bone metastases in the third and fifth lumbar vertebrae. CT of the primary tumor revealed swelling of the left hilar and mediastinal lymph nodes and a nodular shadow in the left B8 (Fig. 3C#4). Pathological examination of the TBLB on the left B8 revealed adenocarcinoma histology. Immunohistochemistry revealed that the tumor was positive for ALK. Based on these findings, a diagnosis with ALK-positive lung adenocarcinoma (cT2bN3M1c: stage IVB) was established. During the examination for lung cancer, there was no exacerbation of dyspnea, and PSL dose was maintained at 15 mg/d. Alectinib (600 mg/d) was initiated as first-line treatment, which resulted in a partial response (Fig. 3D, 10 months after treatment).

## 3. Discussion

This case report describes the development of ALK-positive adenocarcinoma in an elderly patient with RA treated with immunosuppressive agents for RA-OP. Despite long-term follow-up in our hospital, the patient was finally diagnosed with metastatic lung cancer, which resulted in fewer treatment options than in the early stages. Two important issues delayed the diagnosis of lung adenocarcinoma; first, the patient had been treated with immunosuppressive agents, including corticosteroids, against RA-OP for over 10 years; however, there was no evidence of malignancy from cytological and pathological findings by TBLB and brush on the region including where the lung cancer was eventually diagnosed (Figs. 2C#3 and 3C#4). ALK-positive adenocarcinoma occurs in relatively central regions and has a high rate of diagnosis by TBLB and cytology.^[10]^ Second, the chest CT images of RA-OP and lung cancer were similar and corticosteroid treatment resulted in temporary partial reduction of the tumor site. There are many reports of OP during chemotherapy for lung cancer, but pathologically, Romero et al reported that 37% of excised lung cancer specimens had OP adjacent to the malignancy.^[11]^ Corticosteroids were effective against OP masquerading as lung carcinoma.^[9]^

ALK gene rearrangements are present in 3% to 5% non-small cell lung cancers, and generally found in younger patients who are nonsmokers.^[12]^ However, a number of elderly-onset ALK mutations have been reported.^[13]^ In studies of patients with ALK-positive lung cancer, the proportion of elderly patients (≥65 years old) was reported to be 10% to 20%^[14]^ while some studies showed a higher proportion (36–46%).^[13]^ There has only been one reported case of ALK-positive adenocarcinoma in a patient with RA but without OP.^[15]^ The relationship between ALK mutations and RA with OP remains unknown, and chronic inflammation may contribute to the development of lung cancer.

RA patients are known to have a high incidence of lung cancer.^[3]^ It has also been reported that RA activity and severity are associated with lung cancer,^[16]^ suggesting that RA inflammation may influence the mechanism of cancer development.^[17]^ Notably, IL-6/JAK1-induced phosphorylation of STAT3 initiates the transcription of various genes that promote proliferation and prevent apoptosis^[18]^; subsequently, the signaling cascade increases WNT5A, which is upregulated in various cancers, including lung cancer, and is associated with the development of metastasis.^[19]^

Further, lung cancer mimicking organizing pneumonia (LCOP) was reported as a slowly progressive adenocarcinoma with a high proportion of epidermal growth factor receptor mutations, which was almost diagnosed in the early stage.^[20]^ However, these patients with LCOP did not show ALK rearrangement, which is generally diagnosed as advanced lung cancer, as in our case.^[21]^ Taken together, the phenotype of our patient differed from that of patients with LCOP. Recently, some reports have suggested that spectral and perfusion CT can be used to differentiate lung cancer from OP.^[8,22]^

In conclusion, we present a case of advanced lung cancer with ALK mutation during long-term immunosuppressive treatment for RA-OP. Physicians should be aware of the potential development of lung cancer in regions with OP, even after a partial response to corticosteroid therapy.

## Author contributions

KH and TA drafted the manuscript and were responsible for patient care. MK, HT, SN, and YS provided patient care and supervised the revision of the manuscript. All authors read and approved the final manuscript.

**Writing – original draft:** Kazuhito Horie, Takanori Asakura.

**Writing – review & editing:** Keita Masuzawa, Hideki Terai's, Sohei Nakayama, Yusuke Suzuki.

## References

[1] Wasserman A. Rheumatoid arthritis: common questions about diagnosis and management. Rheumatoid Arthritis. 2018;97:8. 29671563

[2] Kadura S Raghu G. Rheumatoid arthritis-interstitial lung disease: manifestations and current concepts in pathogenesis and management. Eur Respir Rev. 2021;30:210011. 34168062 10.1183/16000617.0011-2021 PMC9489133

[3] Simon TA Thompson A Gandhi KK. Incidence of malignancy in adult patients with rheumatoid arthritis: a meta-analysis. Arthritis Res Ther. 2015;17:212. 26271620 10.1186/s13075-015-0728-9 PMC4536786

[4] Zhang L Zhao Q Yuan F. Lung cancer in patients with and without rheumatoid arthritis: a propensity score-matched survival analysis cohort study. Thorac Cancer. 2020;11:1406–13. 32220060 10.1111/1759-7714.13388 PMC7262940

[5] Krupar R Kümpers C Haenel A. Kryptogen organisierende pneumonie versus sekundäre organisierende pneumonie. Pathologe. 2021;42:55–63. 10.1007/s00292-020-00903-8 PMC7812985 33462627

[6] Lohr RH Boland BJ Douglas WW. Organizing pneumonia. Features and prognosis of cryptogenic, secondary, and focal variants. Arch Intern Med. 1997;157:1323–9. 9201006 10.1001/archinte.157.12.1323

[7] Maldonado F Daniels CE Hoffman EA. Focal organizing pneumonia on surgical lung biopsy: causes, clinicoradiologic features, and outcomes. Chest. 2007;132:1579–83. 17890462 10.1378/chest.07-1148

[8] Deng L Zhang G Lin X. Comparison of spectral and perfusion computed tomography imaging in the differential diagnosis of peripheral lung cancer and focal organizing pneumonia. Front Oncol. 2021;11:690254. 34778025 10.3389/fonc.2021.690254 PMC8578997

[9] Huo J Liu C Jin B. Cryptogenic organizing pneumonia masquerading as lung carcinoma: a case report and review of the literature. Exp Ther Med. 2017;15:39–46. 29399056 10.3892/etm.2017.5393 PMC5769272

[10] Kang HJ Lim HJ Park JS. Comparison of clinical characteristics between patients with ALK-positive and EGFR-positive lung adenocarcinoma. Respir Med. 2014;108:388–94. 24361161 10.1016/j.rmed.2013.11.020

[11] Romero S Barroso E Rodriguez-Paniagua M. Organizing pneumonia adjacent to lung cancer frequency and clinico-pathologic features. Lung Cancer. 2002;35:195–201. 11804693 10.1016/s0169-5002(01)00405-6

[12] Gao X Sholl LM Nishino M. Clinical implications of variant ALK FISH rearrangement patterns. J Thorac Oncol. 2015;10:1648–52. 26536196 10.1097/JTO.0000000000000665 PMC4634010

[13] Miyazaki K Sato S Kodama T. Clinicopathological features in elderly ALK-rearranged non-small cell lung cancer patients. In Vivo. 2020;34:2001–7. 32606173 10.21873/invivo.11998 PMC7439864

[14] Bedas A Peled N Maimon Rabinovich N. Efficacy and safety of *ALK* tyrosine kinase inhibitors in elderly patients with advanced *ALK*-positive non-small cell lung cancer: findings from the real-life cohort. Oncol Res Treat. 2019;42:275–82. 30955009 10.1159/000499086

[15] Nakabo S Gochi F Yasuhara Y. Crizotinib may be effective for rheumatoid arthritis: a case report of a rheumatoid arthritis patient with lung cancer. Mod Rheumatol Case Rep. 2017;1:49–53.

[16] Sugimoto N Tanaka E Inoue E. Trends in risks of malignancies in Japanese patients with rheumatoid arthritis: analyses from a 14-year observation of the IORRA cohort. Mod Rheumatol. 2022:roac085. Available at: https://pubmed.ncbi.nlm.nih.gov/35920098/. 35920098 10.1093/mr/roac085

[17] England BR Campany M Sayles H. Associations of serum cytokines and chemokines with the risk of incident cancer in a prospective rheumatoid arthritis cohort. Int Immunopharmacol. 2021;97:107719. 33933845 10.1016/j.intimp.2021.107719 PMC8324526

[18] Heinrich PC Behrmann I Haan S. Principles of interleukin (IL)-6-type cytokine signalling and its regulation. Biochem J. 2003;374(Pt 1):1–20. 12773095 10.1042/BJ20030407 PMC1223585

[19] Morand S Staats H Creeden JF. Molecular mechanisms underlying rheumatoid arthritis and cancer development and treatment. Future Oncol. 2020;16:483–95. 32100561 10.2217/fon-2019-0722

[20] Ichikawa T Saruwatari K Mimaki S. Immunohistochemical and genetic characteristics of lung cancer mimicking organizing pneumonia. Lung Cancer. 2017;113:134–9. 29110840 10.1016/j.lungcan.2017.10.001

[21] Doebele RC Lu X Sumey C. Oncogene status predicts patterns of metastatic spread in treatment-naive nonsmall cell lung cancer: metastatic spread by oncogene in NSCLC. Cancer. 2012;118:4502–11. 22282022 10.1002/cncr.27409 PMC3370097

[22] Yu Y Wang X Shi C. Spectral computed tomography imaging in the differential diagnosis of lung cancer and inflammatory myofibroblastic tumor. J Comput Assist Tomogr. 2019;43:338–44. 30762653 10.1097/RCT.0000000000000840

